# Proliferation of Osteoblasts on Laser-Modified Nanostructured Titanium Surfaces

**DOI:** 10.3390/ma11101827

**Published:** 2018-09-26

**Authors:** Vaclav Babuska, Jan Palan, Jana Kolaja Dobra, Vlastimil Kulda, Michal Duchek, Jan Cerny, Daniel Hrusak

**Affiliations:** 1 Department of Medical Chemistry and Biochemistry, Faculty of Medicine in Pilsen, Charles University, Karlovarska 48, 301 66 Pilsen, Czech Republic; jana.dobra@lfp.cuni.cz (J.K.D.); vlastimil.kulda@lfp.cuni.cz (V.K.); 2 COMTES FHT a.s., Prumyslova 995, 334 41 Dobrany, Czech Republic; jpalan@comtesfht.cz (J.P.); michal.duchek@comtesfht.cz (M.D.); jan.cerny@comtesfht.cz (J.C.); 3 Department of Stomatology, Faculty of Medicine in Pilsen, University Hospital and Charles University, alej Svobody 80, 301 00 Pilsen, Czech Republic; hrusak@fnplzen.cz

**Keywords:** biocompatibility, nanostructured titanium, osteoblasts proliferation, laser treatment

## Abstract

Nanostructured titanium has become a useful material for biomedical applications such as dental implants. Certain surface properties (grain size, roughness, wettability) are highly expected to promote cell adhesion and osseointegration. The aim of this study was to compare the biocompatibilities of several titanium materials using human osteoblast cell line hFOB 1.19. Eight different types of specimens were examined: machined commercially pure grade 2 (cpTi2) and 4 (cpTi4) titanium, nanostructured titanium of the same grades (nTi2, nTi4), and corresponding specimens with laser-treated surfaces (cpTi2L, cpTi4L, nTi2L, nTi4L). Their surface topography was evaluated by means of scanning electron microscopy. Surface roughness was measured using a mechanical contact profilometer. Specimens with laser-treated surfaces had significantly higher surface roughness. Wettability was measured by the drop contact angle method. Nanostructured samples had significantly higher wettability. Cell proliferation after 48 hours from plating was assessed by viability and proliferation assay. The highest proliferation of osteoblasts was found in nTi4 specimens. The analysis of cell proliferation revealed a difference between machined and laser-treated specimens. The mean proliferation was lower on the laser-treated titanium materials. Although plain laser treatment increases surface roughness and wettability, it does not seem to lead to improved biocompatibility.

## 1. Introduction

Titanium is one of the most widely-used implant materials for biomedical applications, thanks to its excellent properties, such as high biocompatibility, non-toxicity, resistance to body fluids, flexibility, and corrosion resistance [1,2,3]. Commercially pure titanium (cpTi) has outstanding biocompatibility but relatively poor strength, whereas titanium alloys, due to their composition and microstructure, have superior strength [4] but, at the same time, they contain potentially toxic or allergenic ingredients [5,6]. Some researchers have focused on new beta-titanium alloys without toxic elements like aluminium or vanadium [7,8].

An effective way to improve the mechanical strength of cpTi is to refine its grain structure. In recent years, much effort has been devoted to important investigations of mechanical properties of ultra-fine grained (UFG) materials. UFG metals exhibit higher strength levels than those with ordinary microstructures. Bulk and fully-dense nanostructured titanium (nTi) can be produced using severe plastic deformation (SPD) methods. UFG materials with a grain size of hundreds of nanometers obtained by SPD were first developed by Valiev [9,10]. There are a number of techniques that lead to extreme grain refinement. One of them is equal channel angular (ECA) pressing. The goal of ECA pressing is to introduce severe plastic strain into a billet of material without changing its cross-section. The same billet can thus be passed through the ECA die repeatedly in order to impart the desired amount of plastic strain. This SPD method has been developed and is now used for producing microstructures with submicron and nanometric grain sizes. Nanostructured titanium produced by SPD processes offers multiple advantages: excellent biocompatibility and extraordinary mechanical properties [11,12].

The concept of biocompatibility is defined as the ability of a material to elicit an appropriate response in the biological environment [13]. The biocompatibility of titanium depends on the spontaneous formation of the oxide layer on the surface of the material. This thin layer protects the implant against corrosion reactions or degradation in a wide range of environmental conditions [14]. The analysis of the biocompatibility typically involves in vitro studies on surface roughness, wettability, composition, crystalline structures, mechanical stability, and cell behavior [15].

The mechanism by which nanostructuring of a material changes its biological properties has not been fully understood yet. In concordance with our results [16], Thirugnanam et al. [17] found significantly higher adhesion of osteoblasts and increased bone formation on nanostructured titanium surfaces when compared to conventional titanium. Since that time, many in vitro as well as in vivo studies have investigated the impact of nanostructured surfaces on the behavior of the surrounding cells [18,19]. Zhou et al. [20] suggested one possible explanation, based on changes in the chemical composition of titanium surface immersed in a simulated body fluid (SBF) with an addition of bovine serum albumin (BSA). The higher levels of calcium, phosphorus and oxygen found on the surface of UFG titanium are beneficial to the bioactivity of the titanium implant [21].

However, good biocompatibility is not the only requirement. To use UFG titanium in vivo, it is also important to improve osseointegration. Long-term success of a dental implant depends on not only osseointegration but also the contact with the surrounding soft tissue [22,23,24]. In clinical applications, implants are expected to exhibit surface bioactivity, promote cell adhesion and accelerate the formation of new bone tissue. It is therefore important to modify the surface to accelerate the integration of the implanted material and the tissue. Numerous methods for modifying implant surfaces have been developed, such as particle blasting [25], chemical etching [26], plasma spraying [27], and anodization [28]. However, these techniques do not produce a highly controllable, morphologically uniform surface topography. Besides, each of these methods may lead to chemical contamination of pure titanium. One of the techniques suitable for roughening an implant surface involves the use of laser. As it obviates the need for direct contact, it also prevents contamination [29,30]. Some authors of recently published studies estimated enhanced proliferation and differentiation on the laser treated titanium surfaces [31]. On the other hand, other studies show no difference or even adverse effects of such surfaces [32]. It remains unclear to what extent the laser treatment is beneficial for cell proliferation.

The aim of this study was to compare the biocompatibilities of nanostructured titanium of two different grades with and without laser treatment. According to our knowledge, there are no studies on the influence of laser modified surface of nTi grade 4 material. The surfaces were characterized by topography, wettability, and roughness and subsequently, tests on biocompatibility were performed. Evaluated material is intended for manufacturing of implants into the bone. For that reason, we decided to use osteogenic cells (osteoblasts) derived from human bones for cell proliferation assays.

## 2. Materials and Methods

### 2.1. Material

All specimens (cylindrical titanium discs, 4.4 mm in diameter and 2.5 mm in height) were manufactured by turning from 5 mm in diameter rods. This was followed by mechanical surface treatment by polishing with abrasive paper to produce smooth surfaces. Eight different versions of the material were used (cpTi2, cpTi4, nTi2 and nTi4, i.e., cpTi and nTi specimens of two titanium grades, and cpTi2L, cpTi4L, nTi2L and nTi4L, i.e., specimens after laser treatment).

**Figure 1:**
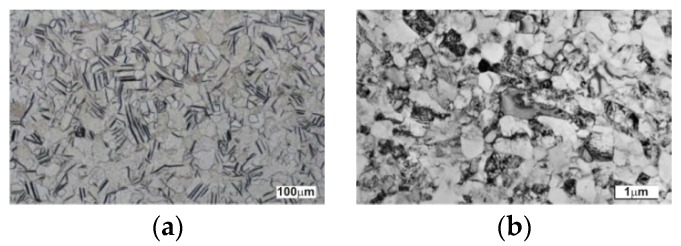
Grain size of (**a**) basic pure titanium (cpTi) and (**b**) nanostructured titanium (nTi) material.

All specimens were taken from grade two and grade four cpTi rods, which are used for the production of dental implants. UFG nanostructured titanium was manufactured by the ECA pressing method on a continuous extrusion machine (Conform 315i, BWE Ltd., Ashford, UK). For further details regarding the ECA pressing technique, the reader is referred to the published work by [33,34,35]. Table 1 gives the basic parameters of cpTi and nTi materials of both grades. The average grain sizes of the basic material and the nanostructured material were 54 µm and 220 nm, respectively (Figure 1). The observation was performed using a transmission electron microscope (JEM-200CX, JEOL Europe, Brussels, Belgium) with an acceleration voltage of 200 kV. Selective electron diffraction was used for determination of the phases. Grain size was measured using the linear intercept method.

Tensile testing was carried out in an electromechanical testing machine (MTS Bionic 25 kN, MTS Systems, Eden Prairie, MN, USA) under quasi-static loading conditions at a constant strain rate of 0.0002 s^−1^ at room temperature. Round bar tensile specimens with a diameter of 3 mm and 5 mm, respectively, were employed. A mechanical extensometer was used to measure the elongation of both testing specimen geometries.

An Nd:YAG laser (Jenoptik Laser Optik, Jena, Germany) with a pulse repetition frequency of 15 kHz and a pulse width of 2 μs was used for ablation of titanium surfaces. It was operated at a sustained wavelength of 1064 nm and a power output of 10 W.

Each sample was cleaned and sterilized before use. The procedure involved incubation in a trypsin solution (0.25% (*w*/*v*) Trypsin-0,53 nM EDTA (ethylenediaminetetraacetic acid) solution, PAA Laboratories GmbH, Pasching, Austria) (30 min, 37 °C), followed by incubation in an ultrasonic bath (20 min, 25 °C), incubation in acetone (30 min, 25 °C), and, at the end, a rinse with 96% ethanol and deionized water. Finally, the implants were sterilized by autoclaving (15 min, 130 °C).

### 2.2. Characterization of Surfaces

Topographic evaluation was performed with a scanning electron microscope (SEM; JSM 6380, JEOL, Tokyo, Japan) in order to compare the eight different implant material surfaces. The secondary electron channel was used for the observation. Each sample was analyzed at 25×, 200×, and 1000× magnification.

The surface roughness of each sample was measured three times using a Surtronic 25 (Taylor Hobson, Leicester, UK) mechanical contact profilometer. It was quantified as the arithmetical mean roughness Ra (defined as the arithmetic average of the absolute values of profile height deviations from the mean line). Surface roughness was measured at a traverse speed of 1 mm/s with a diamond-tipped stylus with a 5 µm radius. An average value from four measurements was recorded as the mean surface roughness for each specimen.

Surface wettability was determined by the drop contact angle method (LeicaS9i, Leica Microsystems, Wetzlar, Germany). Ultrapure water was applied. On each sample, the contact angle was measured 3 s after placing a 2 µL droplet on the surface, which was repeated three times. This test was conducted on five different samples from each group.

### 2.3. Cell Culture

A human foetal osteoblast cell line hFOB 1.19 (ATCC^®^ CRL11372™) was provided by American Type Culture Collection (Manassas, VA, USA), established by Harris et al. [36], was grown in a 1:1 mixture of Ham’s F12 Medium and Dulbecco’s Modified Eagle’s Medium with 2.5 mM L-glutamine (without phenol red) (Gibco, Life Technologies, Paisley, UK) supplemented with 10% (*v*/*v*) foetal bovine serum (FBS) and 0.3 mg/mL geneticin (G418, Serva Electrophoresis GmbH, Heidelberg, Germany). Cells were maintained at 34 °C under 5% CO_2_ in a humidified incubator. Culture media were refreshed as needed.

### 2.4. Cell Proliferation

Cell proliferation after 48 h from plating was assessed by MTT [3-(4,5-Dimethylthiazol-2-yl)-2,5-diphenyltetrazolium bromide] viability and proliferation assay (ScienCellTM Research Laboratories, Carlsbad, CA, USA) according to the manufacturer’s instruction. This assay is based on the conversion of pale yellow tetrazolium MTT to purple formazan crystals, which can be solubilized and then quantified spectrophotometrically.

The samples of implant materials were placed into a 96-well plate (TPP, St. Louis, MO, USA). Cells harvested with trypsin solution from Petri dishes were re-suspended in culture medium and seeded at a density of approximately 250,000 cells/mL onto the top titanium discs in volumes of 20 µL. As a positive control, cells grown directly on a 96-well tissue culture plate were used.

After incubation for 48 h, cells were washed with phosphate-buffered saline (PBS) and incubated with 10 μL MTT (25 mg/mL) solution at 37 °C. After 4 h, 100 μL of MTT solubilisation buffer (equal to the volume of the original culture medium) was added to each well and the insoluble formazan formed was dissolved by pipetting up and down. The absorbance was measured at 570 nm (Nano Drop 1000, Thermo Fisher Scientific, Waltham, MA, USA), subtracting the background absorbance which had been determined at 690 nm.

### 2.5. Cell Staining

Cultured cells were stained with CellTracker™ Green 5-chloromethylfluorescein diacetate (CMFDA) (Molecular Probes, Inc., Eugene, OR, USA) according to the manufacturer’s instructions. Briefly, cells were properly washed with PBS and incubated with 4 μM CMFDA working solution for 30 min at 37 °C. Then, the dye working solution was replaced with fresh, pre-warmed medium and the cells were incubated for another 30 min at 37 °C. Stained cells were analyzed using an Olympus IX 70 fluorescent microscope (Olympus Europa SE, Hamburg, Germany) equipped with Cell R system at 40×, 100×, and 400× magnification. The used objectives were air type.

### 2.6. Statistical Analysis

Five samples from each group were analyzed. To confirm a hypothesis that the data has normal distribution, the test of normality (Kolmogorov-Smirnov) was performed. Statistical comparisons were computed by the two-tailed unpaired *t*-tests. A *p*-value of < 0.05 was considered to indicate a statistically significant difference. Statistical analysis was performed using the SigmaPlot 12.5 software (Systat Software Inc., San Jose, CA, USA).

## 3. Results

### 3.1. SEM Characterization

**Figure 2:**
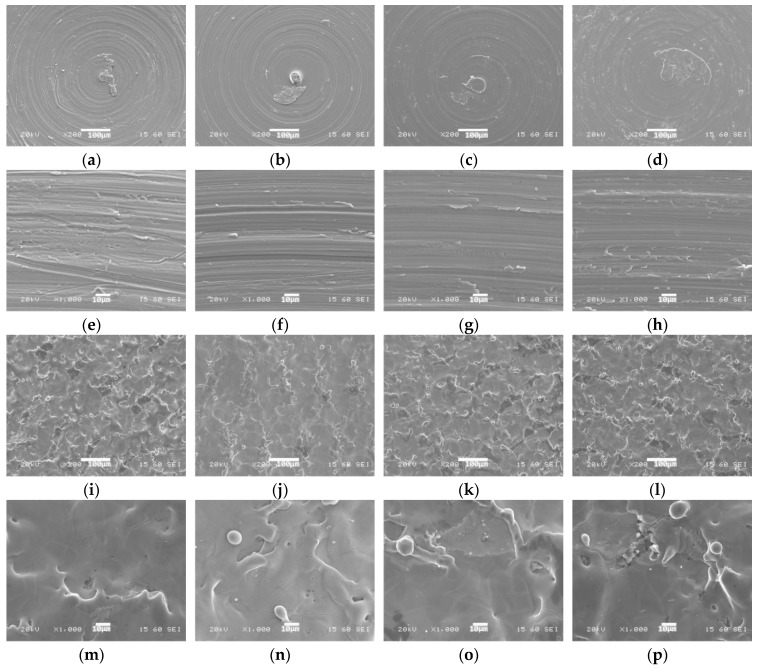
Scanning electron micrographs of the surfaces of samples: (**a**,**e**) cpTi2 and (**i**,**m**) cpTi2L; (**b**,**f**) nTi2 and (**j**,**n**) nTi2L; (**c**,**g**) cpTi4 and (**k**,**o**) cpTi4L; (**d**,**h**) nTi4 and (**l**,**p**) nTi4L. Magnification: 200× (**a**–**d**,**i**–**l**) and 1000× (**e**–**h**,**m**–**p**).

Samples were characterized using SEM. Figure 2 shows scanning electron micrographs of the surfaces of titanium samples. No major differences were found between the individual versions of the material (Figure 2a–d). However, there is a visible difference between machined and laser-treated surfaces of the same material version (Figure 2a–d,i–l).

### 3.2. Roughness and Wettability

The surface roughness quantified by the arithmetical mean roughness Ra is shown in Table 2 for each sample. The Ra values for sandblasted surfaces were between 0.55–0.67 µm and those for laser-treated surfaces were between 2.09–2.51 µm. No significant differences were found between grade 2 and grade 4 basic materials or between grade 2 and grade 4 nanostructured materials. However, machined and laser-treated surfaces showed significantly different roughness values in both grades (*p*-value in Table 2).

**Figure 3:**
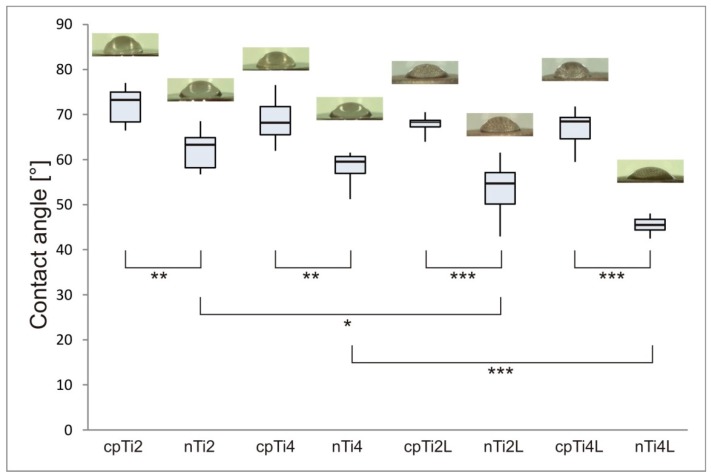
Water contact angle for different surfaces and water droplet morphologies (minimum, 1st quartile, median, 3rd quartile, maximum; *, **, *** denotes statistically significant differences of *p* < 0.05, *p* < 0.01 and *p* < 0.001, respectively).

Figure 3 shows the contact angle of ultrapure distilled water on each type of surface and the corresponding morphology of the water droplet. All nanostructured samples exhibited a significantly higher wettability (lower contact angle) than their plain-structured counterparts. Significantly improved wettability was also found in laser-treated surfaces, but only those of nanostructured samples.

### 3.3. Cell Proliferation

**Figure 4:**
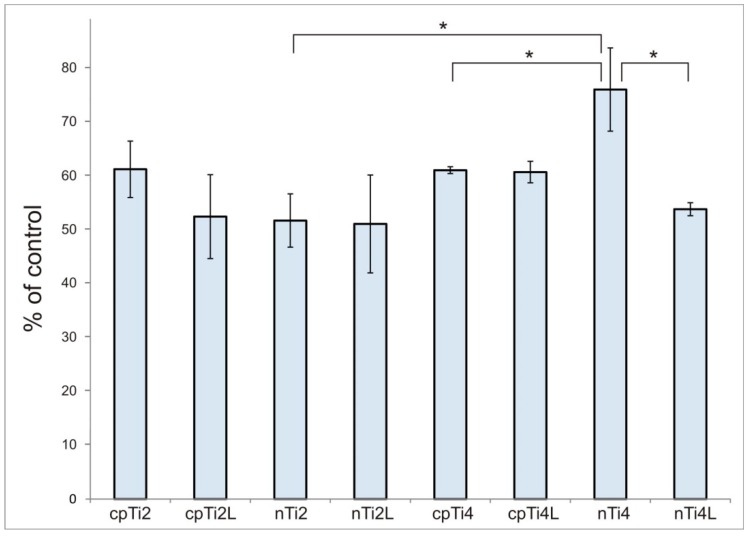
Comparison of the proliferation of osteoblasts on eight versions of titanium surfaces. The data is expressed as % of control. Error bars indicate means ± standard deviations; *, **, *** denotes statistically significant differences of *p* < 0.05, *p* < 0.01 and *p* < 0.001, respectively.

The proliferation data for osteoblasts after culturing for 48 h on different samples is plotted in Figure 4, shown as a number of cells calculated from the MTT calibration curve (the curve itself is not shown).

We found that the proliferation of osteoblasts was higher on nTi4 than on nTi2 (*p* = 0.0200). The comparisons in the other pairs of grades did not exhibit any significant differences.

**Figure 5:**
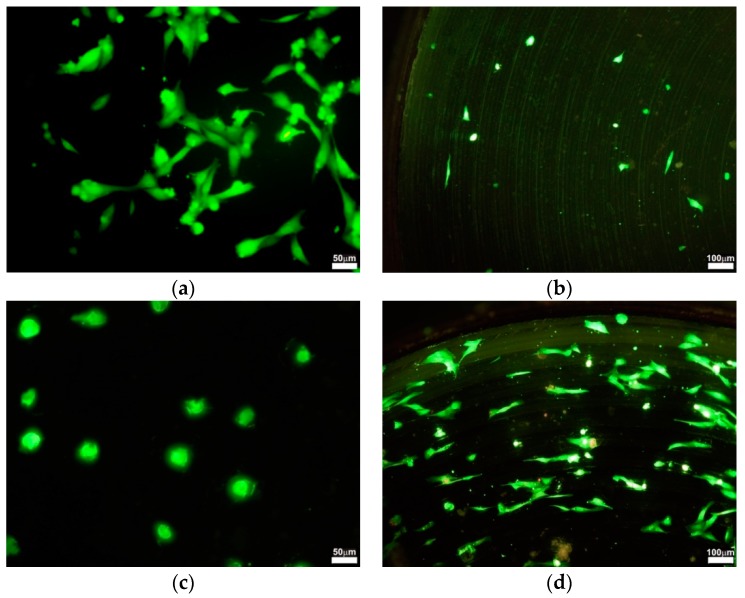
Osteoblasts on different types of studied materials: (**a**) Control cells on the plastic tissue plate; (**b**) Cells on nTi2; (**c**) Cells on laser-treated nTi4L; (**d**) Cells on nTi4.

The analysis of cell proliferation revealed a difference between the behavior of osteoblasts on machined and laser-treated materials. The mean proliferation was lower for all types of laser-treated titanium materials. However, the only significant variation was found between nTi4 and nTi4L (*p* = 0.0159). Figure 5 illustrates the changes in the morphology and number of osteoblasts on different samples. On the tissue culture plastic, no specific orientation was found. By contrast, the cells were aligned to the concentric grooves on the titanium samples.

## 4. Discussion

In recent years, many studies explored the influence of physical and chemical surface characteristics on the biocompatibility of titanium. Many authors have concurred that the biocompatibility of titanium implants depends on the properties of their surfaces [37,38,39]. In this work, we examined how the surface topography and its modification by laser treatment affects the behavior of osteoblastic cells grown on the surfaces of cpTi and UFG nTi of two different grades.

The grain size was shown to be an important factor that influenced not only the strength of material, but also its interactions with cells. Kim et al. [40] proved that ultrafine-grain titanium prepared by the ECA pressing method had better biocompatibility represented by the wettability, cell adhesion, and proliferation of mouse fibroblasts. In our study, samples of nTi4 supported significantly higher proliferation than cpTi4 (*p* = 0.0224). With the other grade, no appreciable difference in proliferation was found between nTi2 and cpTi2 (*p* = 0.1349). Significantly higher proliferation was found in nTi4 than in nTi2 (*p* = 0.0200). Based on these results, one can say that from all the material versions tested, the nTi4 UFG titanium (i.e., the material with the smallest grain size and highest yield and ultimate tensile strength) has the strongest positive impact on the proliferation of the osteoblasts used for this experiment. Our findings appear to be consistent with studies which reported better viability at smaller grain sizes [41,42,43].

It has previously been demonstrated that nano-size irregularities and nano-patterns have an effect on in vitro cell behavior, such as cell proliferation, cell differentiation, and cell activity [44,45]. Martin et al. [46] showed in their study that not only the surface topography, but many other factors as well, are important in the biologic performance of materials. Lincks et al. [47] confirmed the observations that osteoblast-like cells respond in a different manner to both the surface roughness and the material composition. Moreover, it became clear that roughness plays a more important role in determining the cell response than the type of topography, as long as the Ra values can be sensed by the cells. Surface roughness can cause changes in osteoblast proliferation, differentiation and matrix production, but under certain conditions different kinds of osteoblasts may respond in different ways to surface modification [48].

In our work, we further examined the effects of surface roughness alteration by laser treatment on the proliferation of the human osteoblast cell line. Lasers offer high energy levels which can be used for modifying surfaces of various materials and for producing three-dimensional nano and microstructures. They can induce changes in surface roughness and deformation rapidly and effectively [49]. The advantages of lasers include an ability to generate complex features at high resolution [50]. There is no consensus in the literature over the effect of laser treatment on proliferation, mainly due to several variables such as cell type, growth time, and growth conditions. It highlights the importance of this research to our understanding of biocompatibility.

Furthermore, the effect of surface properties on cells is not only the result of surface roughness and topography. The reaction which occurs when the surface of material comes into contact with the media and serum plays an important role as well. As cell culture media and body fluids are water-based, the wettability of the implant affects the attachment of cells to its surface [51]. This initial interaction produces a layer of macromolecules that modify the behavior of the cells [52]. Hydrophilicity can influence the strength and amount of proteins bound to a surface, the conformation and orientation of protein molecules, and the composition of the macromolecular film that forms on the surface by selective adhesion [53,54]. The adsorbed biological molecules can activate receptors located on the outer membrane of cells. The expression of receptors on the cell surface varies with the type of cell and its differentiation stage. Subsequently, these receptors will determine the initial cellular attachment, as well as the short and long-term processes like proliferation and differentiation [55].

Laser treatment is capable of increasing the surface energy of the material, which is an important factor for improving its wettability. Roughening of the surface has previously been shown to improve the wettability and therefore reduce the contact angle [56]. Low contact angles mean high surface energy, which is another factor that can contribute to better cell attachment [57]. Schakenraad et al. [58] demonstrated that surface energy was a governing factor in cell adhesion and proliferation. Melting with laser has a sterilizing effect and provides a contaminant-free surface that can effectively enhance biocompatibility [59,60]. In our study, all laser-treated surfaces exhibited lower contact angles, which indicate higher wettability and surface energy. This can be attributed to capillary-driven water penetration into surface pores.

In this study, the reduction of grain size from 54 µm to 220 nm by ECAP nanostructuring led to significantly lower contact angles, and thus to improved wettability. Figure 3 shows that increased wettability was achieved in both nanostructured materials, nTi2 and nTi4. This is consistent with findings by Sisti et al. [61] that laser-modified titanium surfaces have distinct topographies that provide a larger surface area and enhanced wettability. However, this does not necessarily translate into better biocompatibility.

Laser treatment produces melting pearls and protrusions. Clinkers created by the laser beam can easily fall off. They probably impair biocompatibility and decrease proliferation [62]. Clinkers themselves may also act as stress factors. Moreover, a steep increase in temperature can cause grain growth, which compromises both the biocompatibility and the mechanical properties of UFG materials [63]. In our experiments, laser-treated surfaces exhibited regular troughs of 100 µm diameter (Figure 2i–l). Observation at higher magnifications revealed that short intensive melting and consolidation were caused by the laser beam. Characteristic melting pearls and protrusions can be seen in Figure 2m–p. Cracking, rims, holes and coating fractures were observed on every laser-treated surface.

In order to reduce stress, cells choose to minimize their interfacial contact with the surface, which accordingly reduced cell spreading [64]. After culturing for 48 h, osteoblasts on laser-treated surfaces displayed round morphology with limited spreading (Figure 4c). This finding is consistent with a number of studies showing that no matter what cell types are used, cell spreading is hindered by topographical features on surfaces with convex or concave particles [65]. Another important observation was that in untreated samples, the cells grown on the surface were not randomly distributed. They were arranged in concentric lines following the structure of the surface (Figure 5d).

Although nTi4 exhibited the best proliferation of osteoblasts, it showed significantly poorer values after laser treatment (*p* < 0.0001). In the laser-treated surface of nTi4L, proliferation was about 30% lower. No significant changes in proliferation were found in the other versions of materials after laser treatment. Our results indicate that osteoblasts grow more slowly on a material treated by our laser ablation than on a machined material, regardless of the version of material. In line with our findings, the study by Mariscal-Munoz et al. [66] found the proliferation of mouse calvarial osteoblasts on a laser-treated surface to be lower than on a polished surface.

## 5. Conclusions

Specimens with laser-treated surfaces had significantly higher surface roughness. Nanostructured samples had significantly higher wettability. We found that the highest proliferation of osteoblasts occurred in nTi4 specimens. An analysis of cell proliferation revealed a difference between machined and laser-treated materials. The mean proliferation was lower on laser-treated titanium materials. Although plain laser treatment increases surface roughness and wettability, it does not seem to lead to improved biocompatibility. Considering the important role that the surface of nanostructured titanium plays in its biocompatibility, in vivo studies on osseointegration are needed to evaluate the applicability in medical treatment.

## Figures and Tables

**Table 1:** Mechanical properties of basic and nanostructured bulk materials. σ_0.2_: yield strength, σ_UTS_: ultimate tensile strength, ε_f_: elongation to failure, A_f_: reduction of area.

Parameters	cpTi2	nTi2	cpTi4	nTi4
σ_0.2_ [MPa]	370	970	563	1240
σ_UTS_ [MPa]	480	1043	650	1270
ε_f_ [%]	25	12	24	10
A_f_ [%]	52	54	46	50

**Table 2:** Arithmetical mean roughness Ra values for all types of samples.

Material	cpTi2	cpTi2L	nTi2	nTi2L	cpTi4	cpTi4L	nTi4	nTi4L
Ra [µm]	0.67	2.35	0.60	2.51	0.55	2.09	0.64	2.34
*p*-value	<0.0001		=0.0004		<0.0001		<0.0001	

## References

[1] Banerjee D., Williams J.C. (2013). Perspectives on titanium science and technology. Acta Mater..

[2] Greger M., Cerny M., Kander L., Kliber J. (2009). Structure and properties of titanium for dental implants. Metalurgija.

[3] Ozcan M., Hammerle C. (2012). Titanium as a reconstruction and implant material in dentistry: Advantages and pitfalls. Materials.

[4] Ehtemam-Haghighi S., Cao G.H., Zhang L.C. (2017). Nanoindentation study of mechanical properties of Ti based alloys with Fe and Ta additions. J. Alloy Compd..

[5] Ortiz A.J., Fernandez E., Vicente A., Guirado J.L.C., Ortiz C. (2018). Metallic ions released from stainless steel, nickel-free, and titanium orthodontic alloys: toxicity and DNA damage. Am. J. Orthod. Dentofac. Orthop..

[6] De Morais L.S., Serra G.G., Albuquerque Palermo E.F., Andrade L.R., Müller C.A., Meyers M.A., Elias C.N. (2009). Systemic levels of metallic ions released from orthodontic mini-implants. Am. J. Orthod. Dentofac. Orthop..

[7] Ehtemam-Haghighi S., Liu Y.J., Cao G.H., Zhang L.C. (2016). Phase transition, microstructural evolution and mechanical properties of Ti-Nb-Fe alloys induced by Fe addition. Mater. Des..

[8] Ehtemam-Haghighi S., Liu Y.J., Cao G.H., Zhang L.C. (2016). Influence of Nb on the β → α” martensitic phase transformation and properties of the newly designed Ti-Fe-Nb alloys. Mater. Sci. Eng. C.

[9] Valiev R.Z., Krasilnikov N.A., Tsenev N.K. (1991). Plastic deformation of alloys with submicron-grained structure. Mater. Sci. Eng. A.

[10] Valiev R.Z., Islamgaliev R.K., Alexandrov I.V. (2000). Bulk nanostructured materials from severe plastic deformation. Prog. Mater. Sci..

[11] Valiev R.Z., Langdon T.G. (2006). Principles of equal-channel angular pressing as a processing tool for grain refinement. Prog. Mater. Sci..

[12] Qarni M.J., Sivaswamy G., Rosochowski A., Boczkal S. (2017). Effect of incremental equal channel angular pressing (I-ECAP) on the microstructural characteristics and mechanical behaviour of commercially pure titanium. Mater. Des..

[13] Williams D.F. (2008). On the mechanisms of biocompatibility. Biomaterials.

[14] Bansal R., Singh J.K., Singh V., Singh D.D.N., Das P. (2017). Optimization of oxidation temperature for commercially pure titanium to achieve improved corrosion resistance. J. Mater. Eng. Perform..

[15] Bauer S., Schmuki P., von der Mark K., Park J. (2013). Engineering biocompatible implant surfaces Part I: Materials and surfaces. Prog. Mater. Sci..

[16] Babuska V., Dobra J., Kulda V., Kripnerova M., Moztarzadeh A., Bolek L., Lahoda J., Hrusak D. (2015). Comparison of fibroblast and osteoblast response to cultivation on titanium implants with different grain sizes. J. Nanomater..

[17] Thirugnanam A., Sampath Kumar T.S., Chakkingal U. (2013). Processing and bioactivity evaluation of ultrafine-grained titanium. Ceram. Trans..

[18] Ostrovska L., Vistejnova L., Dzugan J., Slama P., Kubina T., Ukraintsev E., Kubies D., Kralickova M., Hubalek Kalbacova M. (2016). Biological evaluation of ultra-fine titanium with improved mechanical strength for dental implant engineering. J. Mater. Sci..

[19] Babuska V., Moztarzadeh O., Kubikova T., Moztarzadeh A., Hrusak D., Tonar Z. (2016). Evaluating the osseointegration of nanostructured titanium implants in animal models: Current experimental methods and perspectives (review). Biointerphases.

[20] Zhou Q., Wang L., Zou C.H. (2017). Enhanced surface precipitates on ultrafine-grained titanium in physiological solution. Metals.

[21] Burgos-Asperilla L., Garcia-Alonso M.C., Escudero M.L., Alonso C. (2010). Study of the interaction of inorganic and organic compounds of cell culture medium with a Ti surface. Acta Biomater..

[22] Jäger M., Jennissen H.P., Dittrich F., Fischer A., Köhling H.L. (2017). Antimicrobial and osseointegration properties of nanostructured titanium orthopaedic implants. Materials.

[23] Moztarzadeh A., Moztarzadeh O., Kubikova T., Tonar Z., Hrusak D., Zicha A., Babuska V. (2018). Current methods for assessing osseointegration of nanostructured titanium implants. Chem. Listy.

[24] de Siqueira R.A.C., Fontao F.N.G.K., Sartori I.A.D.M., Santos P.G.F., Bernardes S.R., Tiossi R. (2017). Effect of different implant placement depths on crestal bone levels and soft tissue behavior: a randomized clinical trial. Clin. Oral Implants Res..

[25] Lin Y., Huang C.F., Cheng H.C., Shen Y.K. (2013). A modified surface on titanium alloy by micro-blasting process. Adv. Mater. Res..

[26] Nazarov D.V., Zemtsova E.G., Solokhin A., Valiev R.Z., Smirnov V.M. (2017). Modification of the surface topography and composition of ultrafine and coarse grained titanium by chemical etching. Nanomaterials.

[27] Chappuis V., Buser R., Bragger U., Bornstein M.M., Salvi G.E., Buser D. (2013). Long-term outcomes of dental implants with a titanium plasma-sprayed surface: A 20-year prospective case series study in partially edentulous patients. Clin. Implant Dent. Relat. Res..

[28] Kim S.E., Lim J.H., Lee S.C., Nam S.C., Kang H.G., Choi J. (2008). Anodically nanostructured titanium oxides for implant applications. Electrochim. Acta.

[29] Joob-Fancsaly A., Divinyi T., Fazekas A., Daroczi C., Karacs A., Peto G. (2002). Pulsed laser-induced micro- and nanosized morphology and composition of titanium dental implants. Smart Mater. Struct..

[30] Cei S., Legitimo A., Barachini S., Consolini R., Sammartino G., Mattii L., Gabriele M., Graziani F. (2011). Effect of laser micromachining of titanium on viability and responsiveness of osteoblast-like cells. Implant Dent..

[31] Zwahr C., Gunther D., Brinkmann T., Gulow N., Oswald S., Holthaus M.G., Lasagni A.F. (2017). Laser surface pattering of titanium for improving the biological performance of dental implants. Adv. Healthc. Mater..

[32] Ayubianmarkazi N., Karimi M., Koohkan S., Sanasa A., Foroutan T. (2015). An in vitro evaluation of the responses of human osteoblast-like SaOs-2 cells on SLA titanium surfaces irradiated by different powers of CO_2_ lasers. Lasers Med. Sci..

[33] Furukawa M., Horita Z., Nemoto M., Langdon T.G. (2001). Processing of metals by equal-channel angular pressing. J. Mater. Sci..

[34] Palan J., Malecek L., Hodek J., Zemko M., Dzugan J. (2017). Possibilities of biocompatible material production using conform SPD technology. Arch. Mater. Sci. Eng..

[35] Valiev R.Z., Estrin Y., Horita Z., Langdon T.G., Zehetbauer M.J., Zhu Y.T. (2016). Producing bulk ultrafine-grained materials by severe plastic deformation: Ten years later. JOM.

[36] Harris S.A., Enger R.J., Riggs B.L., Spelsberg T.C. (1995). Development and characterization of a conditionally immortalized human fetal osteoblastic cell line. J. Bone Miner. Res..

[37] Jemat A., Ghazali M.J., Razali M., Otsuka Y. (2015). Surface modifications and their effects on titanium dental implants. Biomed. Res. Int..

[38] Hanawa T. (2010). Biofunctionalization of titanium for dental implant. Jpn. Dent. Sci. Rev..

[39] Castner D.G. (2017). Biomedical surface analysis: Evolution and future directions (Review). Biointerphases.

[40] Kim T.N., Balakrishnan A., Lee B.C., Kim W.S., Smetana K., Park J.K., Panigrahi B.B. (2007). In vitro biocompatibility of equal channel angular processed (ECAP) titanium. Biomed. Mater..

[41] Kim T.N., Balakrishnan A., Lee B.C., Dvorankova B., Smetana K., Park J.K., Panigrahi B.B. (2008). In vitro fibroblast response to ultrafine grained titanium produced by a severe plastic deformation process. J. Mater. Sci. Mater. Med..

[42] Estrin Y., Ivanova E.P., Michalska A., Truong V.K., Lapovok R., Boyd R. (2011). Accelerated stem cell attachment to ultrafine grained titanium. Acta Biomater..

[43] Farzin A., Ahmadian M., Fathi M.H. (2013). Comparative evaluation of biocompatibility of dense nanostructured and microstructured Hydroxyapatite/Titania composites. Mater. Sci. Eng. C.

[44] Geblinger D., Addadi L., Geiger B. (2010). Nano-topography sensing by osteoclasts. J. Cell Sci..

[45] Martinez E., Engel E., Planell J.A., Samitier J. (2009). Effects of artificial micro- and nano-structured surfaces on cell behaviour. Ann. Anat..

[46] Martin J.Y., Schwartz Z., Hummert T.W., Schraub D.M., Simpson J., Lankford J., Dean D.D., Cochran D.L., Boyan B.D. (1995). Effect of titanium surface roughness on proliferation, differentiation, and protein synthesis of human osteoblast-like cells (MG63). J. Biomed. Mater. Res..

[47] Lincks J., Boyan B.D., Blanchard C.R., Lohmann C.H., Liu Y., Cochran D.L., Dean D.D., Schwartz Z. (1998). Response of MG63 osteoblast-like cells to titanium and titanium alloy is dependent on surface roughness and composition. Biomaterials.

[48] Györgyey A., Ungvari K., Kecskemeti G., Kopniczky J., Hopp B., Oszko A., Pelsöczi I., Rakonczay Z., Nagy K., Turzo K. (2013). Attachment and proliferation of human osteoblast-like cells (MG-63) on laser-ablated titanium implant material. Mater. Sci. Eng. C.

[49] Trtica M.S., Radak B.B., Gakovic B.M., Milovanovic D.S., Batani D., Desai T. (2009). Surface modifications of Ti6A14V by a picosecond Nd: YAG laser. Laser Part. Beams.

[50] Hindy A., Farahmand F., Tabatabaei F. (2017). In vitro biological outcome of laser application for modification or processing of titanium dental implants. Laser Med. Sci..

[51] Eriksson C., Nygren H., Ohlson K. (2004). Implantation of hydrophilic and hydrophobic titanium discs in rat tibia: cellular reactions on the surfaces during the first 3 weeks in bone. Biomaterials.

[52] Bächle M., Kohal R.J. (2004). A systematic review of the influence of different titanium surfaces on proliferation, differentiation and protein synthesis of osteoblast-like MG63 cells. Clin. Oral Implants Res..

[53] Wilson C.J., Clegg R.E., Leavesley D.I., Pearcy M.J. (2005). Mediation of biomaterial–cell interactions by adsorbed proteins: A review. Tissue Eng..

[54] Andrade J.D., Hlady V. (1986). Protein adsorption and materials biocompatibility - a tutorial review and suggested hypotheses. Adv. Polym. Sci..

[55] Gittens R.A., Scheideler L., Rupp F., Hyzy S.L., Geis-Gerstorfer J., Schwartz Z., Boyan B.D. (2014). A review on the wettability of dental implant surfaces II: Biological and clinical aspects. Acta Biomater..

[56] Koper J.K., Jakubowicz J. (2014). Correlation of wettability with surface structure and morphology of the anodically oxidized titanium implants. J. Biomater. Tissue Eng..

[57] Bodhak S., Bose S., Bandyopadhyay A. (2009). Role of surface charge and wettability on early stage mineralization and bone cell-materials interactions of polarized hydroxyapatite. Acta Biomater..

[58] Schakenraad J.M., Busscher H.J., Wildevuur C.R.H., Arends J. (1986). The influence of substratum surface free energy on growth and spreading of human fibroblasts in the presence and absence of serum proteins. J. Biomed. Mater. Res..

[59] Chikarakara E., Fitzpatrick P., Moore E., Levingstone T., Grehan L., Higginbotham C., Vazquez M., Bagga K., Naher S., Brabazon D. (2015). In vitro fibroblast and pre-osteoblastic cellular responses on laser surface modified Ti-6Al-4V. Biomed. Mater..

[60] Ciganovic J., Stasic J., Gakovic B., Momcilovic M., Milovanovic D., Bokorov M., Trtica M. (2012). Surface modification of the titanium implant using TEA CO_2_ laser pulses in controllable gas atmospheres—Comparative study. Appl. Surf. Sci..

[61] Sisti K.E., de Andres M.C., Johnston D., Almeida-Filho E., Guastaldi A.C., Oreffo R.O.C. (2016). Skeletal stem cell and bone implant interactions are enhanced by LASER titanium modification. Biochem. Biophys. Res. Commun..

[62] Zhang R., Wan Y., Ai X., Wang T., Men B. (2016). Preparation of micro-nanostructure on titanium implants and its bioactivity. Trans. Nonferr. Met. Soc. China.

[63] Zheng C.Y., Nie F.L., Zheng Y.F., Cheng Y., Wei S.C., Valiev R.Z. (2011). Enhanced in vitro biocompatibility of ultrafine-grained titanium with hierarchical porous surface. Appl. Surf. Sci..

[64] Kunzler T.P., Huwiler C., Drobek T., Voros J., Spencer N.D. (2007). Systematic study of osteoblast response to nanotopography by means of nanoparticle-density gradients. Biomaterials.

[65] Gui N., Xu W., Abraham A.N., Myers D.E., Mayes E.L.H., Xia K., Shukla R., Qian M. (2018). A comparative study of the effect of submicron porous and smooth ultrafine-grained Ti-20Mo surfaces on osteoblast responses. J. Biomed. Mater. Res. A.

[66] Mariscal-Muñoz E., Costa C.A.S., Tavares H.S., Bianchi J., Hebling J., Machado J.P.B., Lerner U.H., Souza P.P.C. (2016). Osteoblast differentiation is enhanced by a nano-to-micro hybrid titanium surface created by Yb:YAG laser irradiation. Clin. Oral Investig..

